# A Pediatrician’s Newest Tool: Osteopathic Manipulative Treatment in the Inpatient Setting

**DOI:** 10.51894/001c.143621

**Published:** 2025-10-01

**Authors:** Rasha Khanafer, Ryan Holub-Ward, Matthew Wong, Christine Hart, Chaya Pitman-Hunt

**Affiliations:** 1 College of Osteopathic Medicine Michigan State University; 2 Pediatrics Children’s Hospital of Michigan; 3 College of Osteopathic Medicine Michigan State University

## 05

### INTRODUCTION

Osteopathic manipulative medicine (OMM) is a rapidly expanding and growing field. OMM can be described as a manually guided force that serves to improve physiologic function and support homeostasis. In many cases, OMM is used to treat musculoskeletal dysfunction in an outpatient setting. Evidence for the effectiveness of osteopathic manipulative treatment (OMT) is widely available in adult populations, however, there is a lack of research on the use of OMT in pediatric patients. More specifically, the evidence regarding the utility of OMT in an inpatient pediatric setting is sparse. At the Children’s Hospital of Michigan (CHM), a team of osteopathic physicians sought to create an inpatient pediatric OMT consult service and to describe the patients they treated.

### OBJECTIVES

The objective of this study is to describe the creation of an inpatient consult service and the patients consulted for OMT at CHM and Harper-Hutzel Hospital.

### METHODS

The creation of a pediatric OMT consult service included approval from the Chair and all relevant stakeholders. Six interdisciplinary education sessions were offered to faculty and residents and a Pediatric Grand Rounds was presented to bring awareness. The service began in January 2023. A retrospective analysis of OMT consults in 2023 was conducted. We collected demographics, indications for OMT, consulting team, OMT technique used, and areas treated. A descriptive analysis was done. IRB was approved by our local institution.

### RESULTS

The results of our study demonstrate an overwhelming demand for OMT from various services. Out of 253 total consults in a 12 month period, the diagnoses that lead to the majority of consults include respiratory concerns (29%), feeding difficulties (20%), constipation (14%), and MSK (13%).

### CONCLUSIONS

In a hospital setting, pediatric patients are exposed to various forms of treatment, ranging from medical to surgical to supportive. OMT serves as an additional option for pediatricians to ensure the most optimal outcomes. OMT can be useful in a wide array of diagnoses and serves a clear purpose in pediatric patients. The next step is continuing to educate physicians and families on the utility of OMM to improve somatic and physiologic function in pediatric patients.

evasion and ICI therapy failure. However, targeting EZH2 or KAT2A may significantly increase MHC-I expression, and our work may provide novel targets that improve patient immunotherapy response.

